# Association of METS-IR index with prevalence of gallbladder stones and the age at the first gallbladder stone surgery in US adults: A cross-sectional study

**DOI:** 10.3389/fendo.2022.1025854

**Published:** 2022-10-03

**Authors:** Jin Wang, Junping Yang, Yan Chen, Jing Rui, Maoqi Xu, Mingwei Chen

**Affiliations:** ^1^ Department of General Surgery, The Traditional Chinese Medicine Hospital of Wuhu, Wuhu City, China; ^2^ Department of General Practice, Wuhu City Second People‘s Hospital, Wuhu City, China; ^3^ Department of Endocrinology, The First Affiliated Hospital of Anhui Medical University, Hefei City, China

**Keywords:** gallbladder stones, age at first gallbladder stone surgery, METS-IR index, insulin resistance, metabolic syndrome, prevalence

## Abstract

**Objective:**

The purpose of this study was to assess the correlation between the metabolic score for insulin resistance (METS-IR) index and gallbladder stoneprevalence in US adults, as well as the age at first gallbladder stone surgery.

**Methods:**

A logistic regression analysis, subgroup analysis, and dose-response curve were computed for participants in the 2017-2018 National Health and Nutrition Examination Survey (NHANES) to assess the relationship between the METS-IR index and gallbladder stone prevalence and age at first surgery for gallbladder stones.

**Results:**

This study ultimately included 9452 participants aged >20 years, of whom 534 self-reported a history of gallbladder stones, and after adjusting for all confounders, each unit increase in METS-IR index was associated with a 3.3% increase in gallbladder stone prevalence (OR= 1.033, 95% CI: 1.0258, 1.0403) along with an earlier age at first gallbladder stone surgery 0.26 years (β= -0.26, 95% CI: -0.35, -0.17), stratified analysis showed that increased METS-IR index was associated with increased prevalence of gallbladder stones in all subgroups, and the dose-response curve showed a positive linear correlation between METS-IR index and prevalence of gallbladder stones, while a negative linear correlation was observed between increased METS-IR index and age at first gallbladder stone There was a negative linear correlation between age at surgery.

**Conclusion:**

The METS-IR index has been positively associated with gallbladder stone prevalence, thereby contributing to age at first surgery for gallbladder stones. However, the causal relationship between the METS-IR and gallbladder stones cannot be concluded.

## 1 Introduction

A gallstone is a benign biliary disorder with symptoms such as abdominal discomfort, epigastric pain, nausea, vomiting, and loss of appetite (1). The presence of this condition increases the risk of cholecystitis, pancreatitis, biliary obstruction, and gallbladder cancer (2). Globally, there are ethnic and racial differences when it comes to the prevalence of gallstone disease and gallstone formation. It is estimated that approximately 10% of white adults in Western countries have gallbladder stones(GSD), while the prevalence among African Americans and East Asians is lower than others (1), and the prevalence increases with age, eventually reaching 30% in older populations, regardless of gender, in their 70s (3). Health care costs associated with gallbladder stones are approximately $6 billion per year (4). Over 20 million people suffer from gallbladder stones. Moreover, complications can have serious consequences, increasing health care costs and in some cases even posing an immediate danger to the patient. Therefore, identifying gallbladder stones’ risk factors is especially critical for preventing their development.

Living conditions are improving, but the prevalence of metabolic syndrome remains high. A metabolic syndrome is characterized by an array of metabolic disorders, including obesity (especially abdominal obesity), postprandial hyperglycemia, hypertension, and dyslipidemia. It is widely recognized that an increase in obesity is associated with higher morbidity and mortality from several of the most prevalent diseases in the Western world, including gallstones (5, 6). Metabolic syndrome and gallbladder stones share common risk factors, the most relevant being abdominal obesity and insulin resistance, both of which are associated with increased cholesterol synthesis, excessive biliary cholesterol secretion, and elevated biliary lithogenicity in the body (7). Insulin resistance, one of the central mechanisms of the metabolic syndrome, has been reported to be associated with the development of gallbladder stones (8). In peripheral tissues, insulin sensitivity is currently assessed by the high insulin normoglycemic clamp (HEC) (9). Due to its complexity, time, and resource consumption, insulin resistance is often assessed using simpler metrics. A novel insulin resistance (IR) metric was developed in 2018 as a simple, reliable, and reproducible predictor of IR (6). It can be hypothesized that the METS-IR index relates to gallbladder stones, since it has been proposed as a marker of IR. It has not been previously evaluated whether METS-IR index is associated with gallbladder stones. As such, in this study we examined the METS-IR index’s role in gallbladder stone development in the adult United States population.

## 2 Materials and methods

### 2.1 Study population

**Figure 1:**
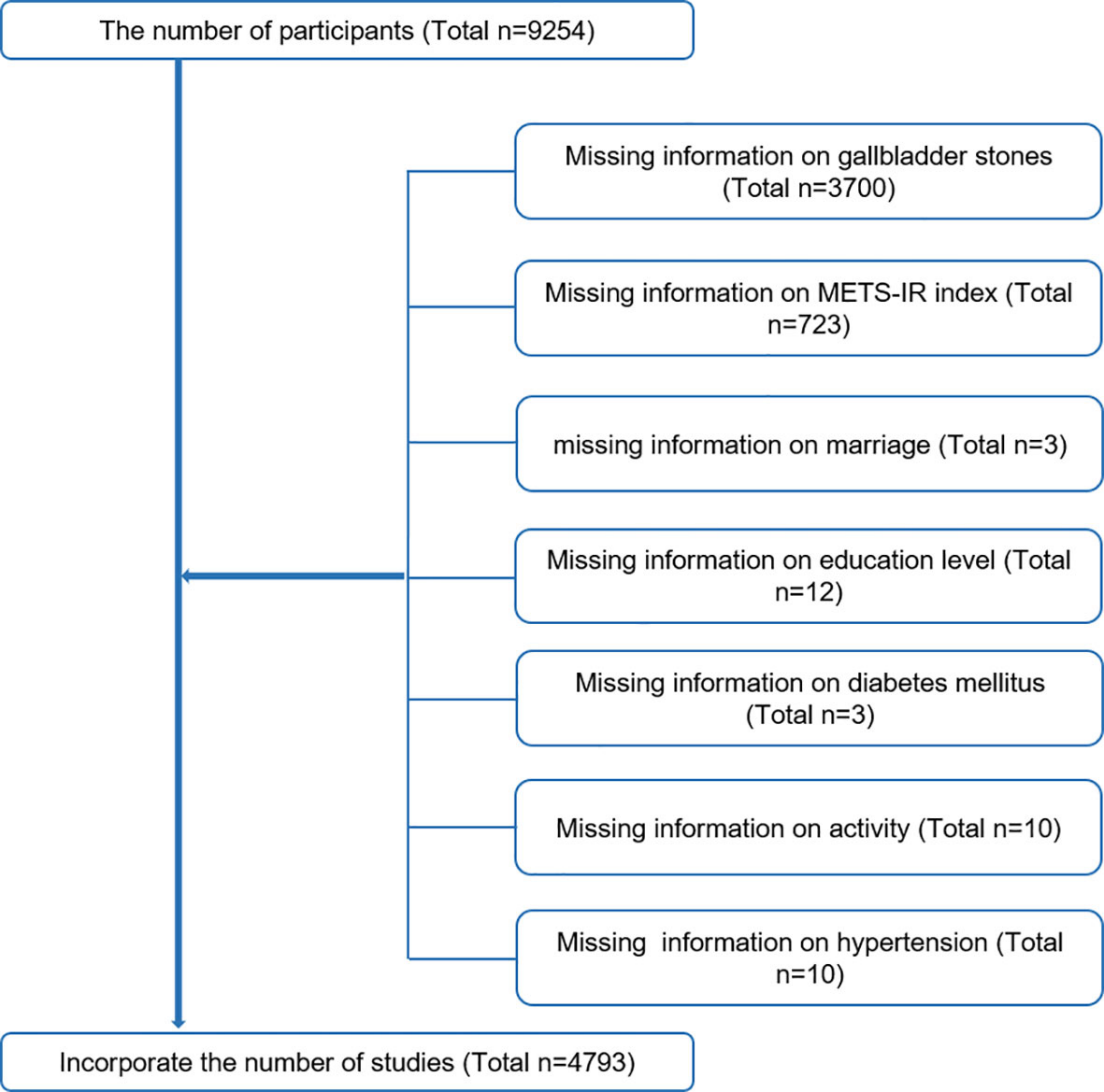
Sample selection flowchart from NHANES 2017–2018.

This study used clinical data from the NHANES from 2017-2018 to determine baseline clinical variables. Our data included information on participants who explicitly answered whether they had gallbladder stones and their age when they had their first gallbladder stone surgery. A total of 9254 people participated in the survey. Exclusion criteria were as follows (**Figure 1**). Finally a total of 4793 cases were included in this study, including 534 self-reported gallbladder stone history.

### 2.2 Data collection and definition

METS-IR index was designed as an exposure variable. METS-IR= Ln[(2 × fasting glucose) + fasting triglycerides) × body mass index]/[Ln(high-density lipoprotein cholesterol)]. An automated biochemical analyzer was used to determine triglyceride and fasting blood glucose levels enzymatically. With the Roche Cobas 6000 chemistry analyzer and the Roche Modular P, serum triglyceride concentrations were determined. Gallbladder stones and age at the time of first gallbladder stone surgery were assessed *via* questionnaires, including “Ever been told you have gallbladder stones?” and “Age when first had gallbladder surgery?.”. The results obtained by intersecting the participants who answered the age of the first gallbladder surgery with those who answered that they had gallbladder stones were considered to be the participants who had the first surgery for gallbladder stones. The occurrence of gallbladder stones and age at first gallbladder stone surgery were designed as outcome variables.

Multivariable adjusted models have been constructed to assess whether potential confounding factors may be involved in the association between METS-IR index and gallbladder stones. Covariates in our study included sex (male/female), age (years), race, education level, poverty to income ratio (PIR), marital status (married or living with partner/single), alcohol consumption (drinking or not), physical activity (vigorous/moderate/below moderate), cholesterol level (mg/dl), smoking status (smoking or not), hypertension, diabetes mellitus, and dietary intake factors, including energy intake, fat intake, sugar intake, and water intake, all participants underwent two 24-hour dietary recalls in years 2017-2018, and the average consumption of the two recalls will be used in our analyses. The details of the measurement procedures for the study variables can be found at http://www.cdc.gov/nchs/nhanes/. All NHANES protocols were implemented in accordance with the U.S. Department of Health and Human Services (HHS) Human Research Subject Protection Policy and were reviewed and standardized annually by the NCHS Research Ethics Review Committee. All subjects who participated in the survey signed informed consent forms. All data in this study were released free of charge by NHANES without additional authorization or ethical review.

### 2.3 Statistical methods

To illustrate the complex, multistage sampling design used in selecting a representative noninstitutionalized U.S. population, the sampling weights, stratification, and clustering provided in the NHANES study were applied to all statistical analyses. To exclude the problem of cointegration, we used the cointegration test, when VIF greater than 5 was considered to have cointegration problem. Continuous variables were represented with weighted survey means and 95% confidence intervals, and categorical variables were represented with weighted survey means and 95% confidence intervals. The presence of gallbladder stones and the time to first gallbladder stone surgery were investigated in three different models using multiple logistic regression analyses based on the guidelines (10). In model 1, no adjustment for covariates was made. Model 2 was adjusted for sex, age and race, marital status, and education level. Model 3 was adjusted for all variables. An analysis of the relationship between METS-IR index and gallbladder stone prevalence and age at first surgery was carried out using smoothed curve fitting (penalized spline method) and generalized additive model (GAM) regression. In cases where a nonlinear relationship is present, an inflection point value is derived by a likelihood ratio test. Next, multiple regression analyses were conducted stratified by sex, age, race, hypertension, and diabetes. P < 0.05 was considered statistically significant. All analyses were performed using Empower software www.empowerstats.com; X&Y Solutions, Inc., Boston, MA, USA) and R version 4.0.2 (http://www.R-project.org, The R Foundation).

## 3 Results

Listed below are the basic demographic characteristics of the participants (**Table 1**). METS-IR index was 50.10 (48.08,52.12) in the gallbladder stone group, which was higher than 43.81 (42.89,44.74) in the normal group, p < 0.0001. Compared to the normal group, the age at the time of the disease, the proportion of women, hypertension, and diabetes were significantly higher in the gallbladder stone population than in the normal group (P < 0.05).

**Table 1:** Baseline characteristics of participants, weighted.

Characteristic	Nonstone formers (n = 4259)	Stone formers (n = 534)	P-value
Age (years)	47.25 (46.05,48.46)	56.47 (55.38,57.55)	<0.0001
Serum Cholesterol (mg/dl)	189.88 (186.41,193.36)	187.70 (182.31,193.10)	0.3459
METS-IR Index	43.81 (42.89,44.74)	50.10 (48.08,52.12)	<0.0001
Gender			<0.0001
Male	50.95 (48.81,53.08)	26.02 (21.40,31.25)	
Female	49.05 (46.92,51.19)	73.98 (68.75,78.60)	
Race			0.0526
Mexican American	16.13 (12.16,21.07)	13.11 (9.56,17.71)	
White	62.23 (56.41,67.71)	70.05 (61.46,77.43)	
Black	11.20 (8.01,15.44)	7.04 (4.98,9.87)	
Other Race	10.45 (7.98,13.57)	9.80 (5.74,16.23)	
Education Level			0.8148
Less than high school	11.28 (9.60,13.21)	10.13 (7.32,13.84)	
High school	27.03 (23.55,30.81)	28.36 (22.25,35.38)	
More than high school	61.69 (57.06,66.12)	61.51 (55.77,66.95)	
Marital Status			0.3617
Cohabitation	63.31 (60.52,66.02)	60.75 (54.14,66.98)	
Solitude	36.69 (33.98,39.48)	39.25 (33.02,45.86)	
Alcohol			0.014
Yes	6.94 (5.38,8.91)	3.97 (1.93,7.97)	
No	8.08 (7.18,9.08)	3.76 (1.84,7.52)	
Unclear	84.98 (83.09,86.69)	92.27 (86.73,95.62)	
High Blood Pressure			<0.0001
Yes	30.29 (27.63,33.08)	49.67 (43.52,55.82)	
No	69.71 (66.92,72.37)	50.33 (44.18,56.48)	
Diabetes			<0.0001
Yes	10.07 (8.92,11.34)	22.81 (18.16,28.25)	
No	89.93 (88.66,91.08)	77.19 (71.75,81.84)	
Smoked			0.0603
Yes	41.75 (38.99,44.56)	48.22 (39.75,56.80)	
No	58.25 (55.44,61.01)	51.78 (43.20,60.25)	
Physical Activity			0.0002
Never	23.54 (21.46,25.75)	29.00 (23.78,34.85)	
Moderate	28.39 (25.38,31.61)	35.61 (28.51,43.40)	
Vigorous	48.07 (45.99,50.17)	35.39 (29.54,41.71)	
PIR			0.0166
<1.3	17.78 (16.24,19.43)	16.80 (12.43,22.32)	
≥1.3,<3.5	30.97 (27.28,34.92)	40.13 (32.33,48.45)	
≥3.5	40.97 (36.62,45.47)	32.07 (27.01,37.60)	
Unclear	10.28 (8.37,12.56)	11.00 (8.14,14.69)	
Total Kcal			0.0091
Lower	37.71 (35.66,39.80)	47.10 (40.42,53.89)	
Higher	45.08 (42.80,47.38)	39.92 (33.35,46.88)	
Unclear	17.21 (14.86,19.85)	12.98 (9.89,16.84)	
Total Sugar			0.8265
Lower	34.10 (31.72,36.56)	35.98 (30.18,42.22)	
Higher	35.00 (32.21,37.89)	34.23 (27.46,41.70)	
Unclear	30.91 (27.73,34.28)	29.80 (24.89,35.22)	
Total Water			0.012
Lower	37.13 (35.24,39.05)	45.64 (39.02,52.42)	
Higher	45.66 (43.32,48.02)	41.38 (35.12,47.94)	
Unclear	17.21 (14.86,19.85)	12.98 (9.89,16.84)	
Total Fat			0.012
Lower	37.13 (35.24,39.05)	45.64 (39.02,52.42)	
Higher	45.66 (43.32,48.02)	41.38 (35.12,47.94)	
Unclear	17.21 (14.86,19.85)	12.98 (9.89,16.84)	

For continuous variables: survey-weighted mean (95% CI), P-value was by survey-weighted linear regression.

For categorical variables: survey-weighted percentage (95% CI), P-value was by survey-weighted Chi-square test.

### 3.1 A higher METS-IR index is associated with a higher prevalence of gallbladder stones

According to the results of collinearity test, the VIF values of all covariates are less than 5, there is no collinearity problem, and all of them are included in the regression model. There was a positive correlation between METS-IR index and gallbladder stones (**Table 2**). According to the fully adjusted model (model 3) (OR=1.033, 95% CI: 1.0258, 1.0403), there was a 3.3% increase in gallbladder stoneprevalence for every unit increase in METS-IR index. For sensitivity analysis, we converted the METS-IR index into a categorical variable (tertile). The odds ratio for gallbladder stoneprevalence was greater by 1.9859 in Tertile 3 (OR=2.9859, 2.2853, 3.9014) than in Tertile 1, the lowest METS-IR index tertile.

**Table 2:** Analysis between METS-IR index with gallbladder stone prevalence.

Characteristic	Model 1 OR (95%CI)	Model 2 OR (95%CI)	Model 3 OR (95%CI)
METS-IR Index	1.0315 (1.0253, 1.0377)	1.0379 (1.0311, 1.0447)	1.0330 (1.0258, 1.0403)
Categories			
Tertile 1	1	1	1
Tertile 2	1.8199 (1.4079, 2.3525)	1.9242 (1.4748, 2.5104)	1.8026 (1.3766, 2.3604)
Tertile 3	2.9243 (2.2957, 3.7250)	3.5126 (2.7205, 4.5354)	2.9859 (2.2853, 3.9014)

Model 1=no covariates were adjusted.

Model 2=Model 1+age, gender, race education, marital status were adjusted.

Model3=Model 2+, diabetes, blood pressure, PIR, total water, total kcal, total sugar, smoked, physical activity, alcohol use, serum cholesterol were adjusted.

### 3.2 Analysis of the dose-response and threshold effects of METS-IR on the prevalence of gallbladder stones

**Figure 2:**
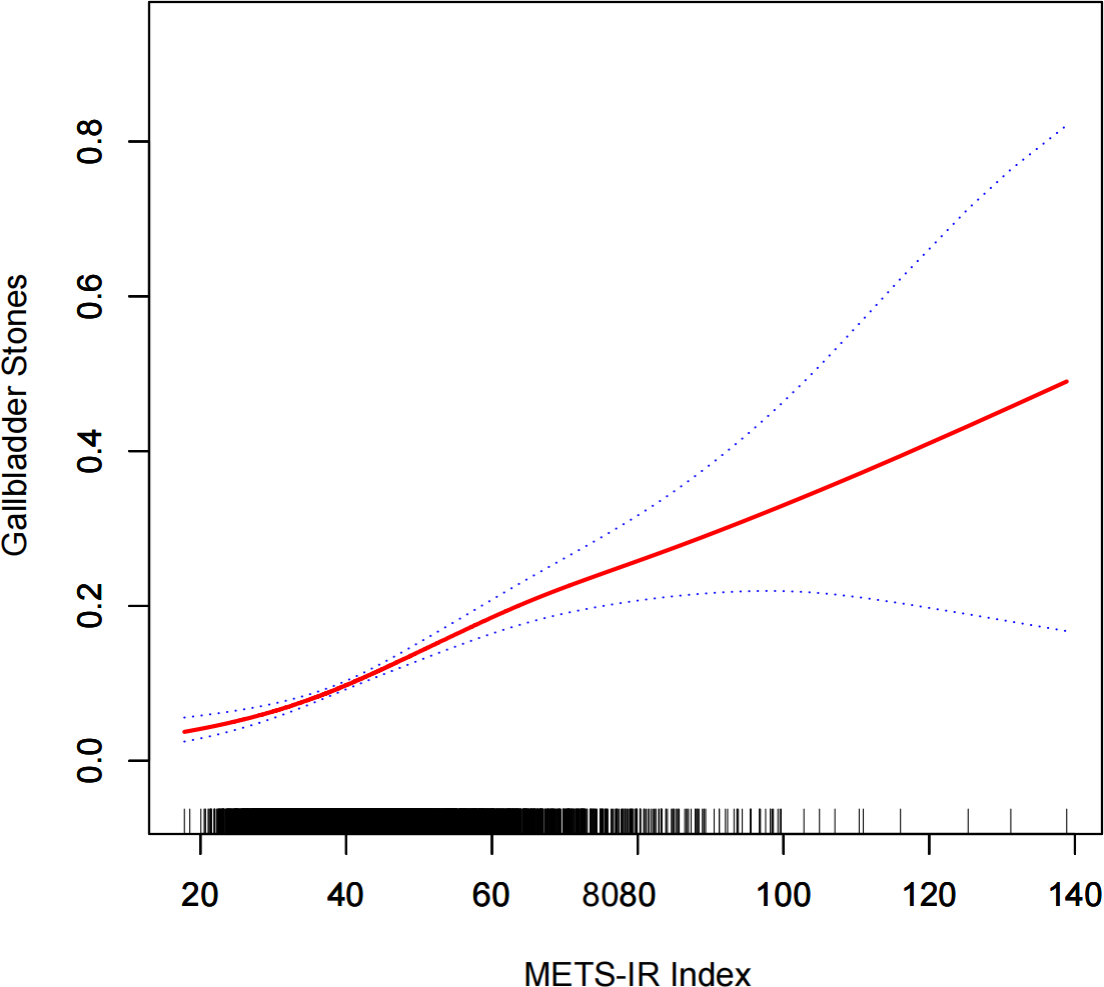
Densitometric dose-response relationship between METS-IR index and gallbladder stone prevalence. The area between the upper and lower dashed lines is indicated as the 95% CI. the red line is connected by the magnitude of the METS-IR index into a continuous line. Adjustments were made for all covariates except for effect modifiers.

An additive generalized model and smoothed curve fitting were used to investigate the relationship between METS-IR index and gallbladder stoneprevalence. In **Figure 2**, we found a linear correlation between the METS-IR index and gallbladder stonesprevalence.

### 3.3 Subgroup analysis

In order to assess the robustness of the association between METS-IR index and gallbladder stoneprevalence, subgroup analyses were conducted. In the whole subgroup analysis, although the METS-IR index showed a positive increase with increasing prevalence of gallbladder stones in all subgroups, it still had different risk effects in different subgroups (**Table 3**). In the gender subgroup, elevated METS-IR was associated with a higher prevalence of gallbladder stones in female(OR=1.0382,95%CI:1.0294, 1.0470) patients compared to males(OR=1.0206,95%CI:1.0064, 1.0350). In the age subgroup, elevated METS-IR was found to be associated with a higher prevalence of gallbladder stones in the younger age subgroup. In the hypertensive and diabetic subgroups, elevated METS-IR was associated with a higher prevalence of gallbladder stones than in the non-hypertensive and non-diabetic groups. Finally, in the racial stratification, we found that elevated METS-IR was associated with a higher prevalence of gallbladder stones in white and other populations.

**Table 3:** Subgroup analysis between METS-IR index with gallbladder stone prevalence.

Characteristic	Model 1 OR (95%CI)	Model 2 OR (95%CI)	Model 3 OR (95%CI)
Subgroup analysis stratified by gender			
Male	1.0226 (1.0110, 1.0344)	1.0283 (1.0154, 1.0414)	1.0206 (1.0064, 1.0350)
Female	1.0378 (1.0302, 1.0455)	1.0418 (1.0336, 1.0501)	1.0382 (1.0294, 1.0470)
Subgroup analysis stratified by age (years)			
20-39	1.0458 (1.0328, 1.0590)	1.0523 (1.0384, 1.0664)	1.0499 (1.0344, 1.0655)
40-59	1.0368 (1.0257, 1.0480)	1.0386 (1.0270, 1.0503)	1.0313 (1.0190, 1.0438)
60-80	1.0231 (1.0131, 1.0332)	1.0238 (1.0134, 1.0343)	1.0164 (1.0052, 1.0278)
Subgroup analysis stratified by hypertension			
YES	1.0262 (1.0174, 1.0352)	1.0341 (1.0243, 1.0441)	1.0265 (1.0161, 1.0370)
NO	1.0305 (1.0214, 1.0396)	1.0381 (1.0282, 1.0481)	1.0365 (1.0261, 1.0469)
Subgroup analysis stratified by diabetes			
YES	1.0227 (1.0112, 1.0344)	1.0320 (1.0182, 1.0459)	1.0299 (1.0155, 1.0444)
NO	1.0292 (1.0216, 1.0369)	1.0359 (1.0277, 1.0442)	1.0346 (1.0261, 1.0432)
Subgroup analysis stratified by race			
Mexican American	1.0238 (1.0091, 1.0386)	1.0341 (1.0176, 1.0509)	1.0273 (1.0100, 1.0450)
White	1.0322 (1.0230, 1.0416)	1.0399 (1.0298, 1.0500)	1.0351 (1.0241, 1.0463)
Black	1.0331 (1.0200, 1.0463)	1.0302 (1.0164, 1.0441)	1.0289 (1.0142, 1.0438)
Other Race	1.0329 (1.0157, 1.0504)	1.0449 (1.0261, 1.0641)	1.0361 (1.0153, 1.0572)

Model 1=no covariates were adjusted.

Model 2=Model 1+age, gender, race education, marital status were adjusted.

Model3=Model 2+, diabetes, blood pressure, PIR, total water, total kcal, total sugar, smoked, physical activity, alcohol use, serum cholesterol were adjusted.

The subgroup analysis was stratified by sex, race, age, diabetes and hypertension, not adjusted for the stratification variable itself.

### 3.4 METS-IR may be associated with earlier age at first gallbladder stone surgery

As a result of fully adjusted model 3, we found that each 1-unit increase in METS-IR index elevation was associated with 0.26 years earlier age at first gallbladder stone surgery (β= -0.26, 95% CI: -0.35, -0.17) (**Table 4**).

**Table 4:** Analysis between METS-IR index with age at the first gallbladder stone operation.

Characteristic	Model 1 β (95%CI)	Model 2 β (95%CI)	Model 3 β (95%CI)
METS-IR Index	-0.24 (-0.33, -0.15)	-0.25 (-0.34, -0.17)	-0.26 (-0.35, -0.17)

Model 1=no covariates were adjusted.

Model 2=Model 1+age, gender, race education, marital status were adjusted.

Model3=Model 2+, diabetes, blood pressure, PIR, total water, total kcal, total sugar, smoked, physical activity, alcohol use, serum cholesterol were adjusted.

### 3.5 Analysis of the dose response and threshold effect of METS-IR on age at first gallbladder stone surgery

**Figure 3:**
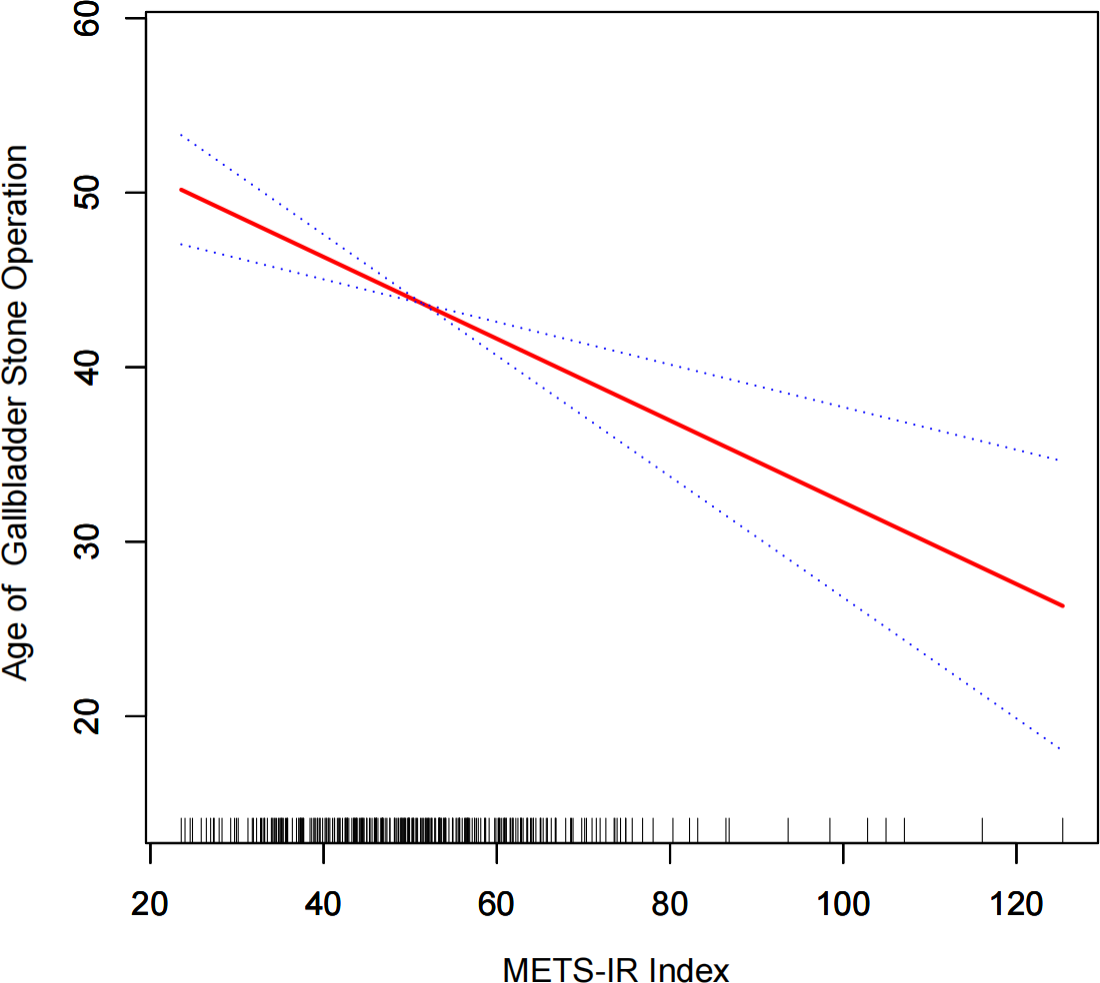
Dense dose-response relationship between METS-IR index and age at the time of first gallbladder stone surgery. The area between the upper and lower dashed lines is indicated as the 95% CI. the red line is connected by the magnitude of the METS-IR index into a continuous line. Adjustments were made for all covariates except for effect modifiers.

Using a generalized additive model and smoothed curve fitting, we examined the relationship between METS-IR index and the age at first gallbladder stone surgery. **Figure 3** shows that METS-IR index and age at first gallbladder stone surgery are linearly correlated (negative).

## 4 Discussion

In a representative sample of US adults, this study demonstrated that METS-IR index increases were associated with an increase in gallbladder stoneprevalence of 3.3% for each unit increase in METS-IR index. Additionally, we found that elevated METS-IR was associated with an earlier age at first gallbladder stone surgery, a study that has never been published before. As a chronic disease causing morbidity, quality of life, and medical costs, gallbladder stones are especially important to prevent. These pressures continue to increase worldwide. Preventing gallbladder stones can be improved by finding populations that are adaptable to the METS-IR index. Consequently, we performed a sensitivity subgroup analysis and found that the METS-IR index is positively correlated with the prevalence of gallbladder stones in almost every population. However, when we performed a sensitivity subgroup analysis, we found that the METS-IR index was positively associated with the prevalence of gallbladder stones in almost all populations, but there were still subtle differences in the different subgroups. In fact, age as a risk factor for cholelithiasis remains controversial. Many studies have reported that age is the main risk factor for gallstones (11, 12), but some studies have found that the effects of metabolic syndrome and obesity on gallstones are stronger in younger participants (13). Therefore, further studies on the effect of age on gallbladder stones are still needed. As for the effect of gender on gallbladder stones, our results are consistent with previous studies reporting that in female patients (1, 14), more severe insulin resistance or metabolic syndrome is associated with a higher incidence of gallbladder stones. According to a Korean study (15), insulin resistance is associated with gallstones in non-hypertensive and non-diabetic individuals. Chen et al. (16) found that elevated METS-IR index was associated with increased asthma prevalence in non-hypertensive and non-diabetic populations, while Shen et al. (6) had similar findings in their study of METS-IR index and kidney stone prevalence. Although the above two studies were conducted on different subjects, they also reflect that our findings may be correct. There was a significant correlation between METS-IR index usage and age, sex, race, hypertension, and diabetes subgroups, indicating a high prevalence of METS-IR index use among gallbladder stone sufferers.

There are millions of people in developed countries suffering from gallstone disease. About 10-15% of the population is thought to be affected by the disease. It can occur at any age and in any gender, but women and people over 50 years of age tend to be more affected (17, 18). Gallbladder stones are most commonly treated with cholecystectomy, but about one-third of the population has surgical complications that persist for a long time and adversely affect their health (19, 20), such as dyspepsia and postoperative pain. When primary prevention strategies are identified to prevent gallbladder stones from forming, clinical outcomes may be significantly improved in patients with gallbladder stones. In this study, METS-IR was also found to be an important factor to consider when determining whether a gallbladder stone has to be surgically removed. According to our results, for every 1 unit increase in METS-IR index, the age at first gallbladder stone surgery will be advanced by 0.26 years. Smoothing curve fitting even showed a linear negative correlation of METS-IR for age at first gallbladder stone surgery. This finding is promising and has not yet been reported. We hypothesize that treatment and management of IR at a younger age may be beneficial in improving or reducing gallbladder stone occurrence. The veracity of this result may be limited by the sample size and needs to be further confirmed by a multicenter large sample prospective study.

The METS-IR index was first reported in 2018 as a practical and intuitive predictor of IR for clinical decision-making (6, 16). It has now been shown that IR can cause gallbladder stones to develop or become exacerbated in many studies, and visceral obesity and hepatic insulin resistance may be central to promoting cholesterol bile supersaturation and gallstone formation (21). Studies show that insulin resistance causes cholesterol supersaturated bile to be produced in high-risk Hispanic populations, resulting in altered gallbladder function leading to gallbladder stones (8). Gallbladder stone formation may also be related to insulin resistance in postmenopausal Korean women with abdominal obesity (22). The formation of cholesterol gallstones was significantly predisposed to mice with isolated hepatic insulin resistance (LIRKO mice), which are deficient in insulin receptors in the liver (23). Another *in vivo* experiment showed that mice with high protein and high quality diets developed sludge and gallstones more quickly (7). According to one study, pioglitazone is an antidiabetic that prevents gallstone formation, liver damage, and gallbladder damage, and guinea pigs treated with pioglitazone showed beneficial changes in the biliary cholesterol and bile acids, blood glucose, insulin, and lipid distribution (24). All of the above reports suggest that IR plays a key role in the development of gallbladder stones, and the fact that METS-IR index is positively correlated with IR levels could explain the association of higher METS-IR indexes with increased prevalence of gallbladder stones.

It has several advantages, including the fact that NHANES represents the U.S. population and follows a rigorous study protocol with extensive quality assurance and quality control. Furthermore, our results were adjusted for confounding covariates to ensure that they would be reliable and applicable to a wider variety of individuals. It is important to note, however, that our study is not without limitations. Since our study was based on the NHANES database, which is a cross-sectional study, we were unable to establish a causal link between the METS-IR index and gallbladder stones. As a second limitation, gallbladder stones were diagnosed based on a questionnaire, which is prone to recall bias. Finally, the database did not provide detailed clinical variables, such as medication history and specific stone composition. While the present study has some limitations, it was able to demonstrate a correlation between METS-IR index and the prevalence of gallbladder stones and the age at which gallbladder stones were first discovered.

## 5 Summary

A higher METS-IR index is associated with an earlier prevalence of gallbladder stones and an earlier age of first gallbladder stone surgery. Although a causal relation between the relationship is not established, treating and giving IR at a young age may improve or minimize the occurrence of gallbladder stones and postpone the age of first gallbladder stone operation.

## Data availability statement

The datasets presented in this study can be found in online repositories. The names of the repository/repositories and accession number(s) can be found below: https://www.cdc.gov/nchs/nhanes.

## Ethics statement

The studies involving human participants were reviewed and approved by The NCHS Research Ethics Review Committee approved the NHANES. The patients/participants provided their written informed consent to participate in this study.

## Author contributions

Data analysis and manuscript writing: JW, MC. Study design and statistical advice: JW, JY, YC. Manuscript editing: JY, YC, JR, MX. Validation and review: YC, JR, MX. Quality control: MC. All authors agreed on the journal to which the article was to be submitted and agreed to take responsibility for all aspects of the work.

## Funding

This work was supported by the Natural Science Foundation of Anhui Province (2108085MH269).

## Acknowledgments

We would like to thank all NHANES participants and staff. We are also grateful to Dr Xudong Shen for providing design ideas and statistical methodology advice.

## Conflict of interest

The authors declare that the research was conducted in the absence of any commercial or financial relationships that could be construed as a potential conflict of interest.

## Publisher’s note

All claims expressed in this article are solely those of the authors and do not necessarily represent those of their affiliated organizations, or those of the publisher, the editors and the reviewers. Any product that may be evaluated in this article, or claim that may be made by its manufacturer, is not guaranteed or endorsed by the publisher.

## Abbreviations

NHANES, National Health and Nutrition Examination Survey; BMI, body mass index; PIR, ratio of family income to poverty; NCHS, National Center for Health Statistics; CI, confidence interval; OR, odds ratio; MetS, metabolic syndrome; IR, insulin resistance; TG, Triglyceride; TC, Cholesterol; FPG, fasting plasma glucose.
